# Application of Quantum Dot Interface Modification Layer in Perovskite Solar Cells: Progress and Perspectives

**DOI:** 10.3390/nano12122102

**Published:** 2022-06-18

**Authors:** Yankai Zhou, Xingrui Luo, Jiayan Yang, Qingqing Qiu, Tengfeng Xie, Tongxiang Liang

**Affiliations:** 1 Engineering Research Center for Hydrogen Energy Materials and Devices, College of Rare Earths, Jiangxi University of Science and Technology, 86 Hong Qi Road, Ganzhou 341000, China; zyk1904500652@163.com (Y.Z.); yangjy021129@163.com (J.Y.); txliang@jxust.edu.cn (T.L.); 2 Faculty of Materials Metallurgy and Chemistry, Jiangxi University of Science and Technology, 86 Hong Qi Road, Ganzhou 341000, China; luoxingrui2002@163.com; 3 College of Chemistry, Jilin University, Changchun 130012, China; xietf@jlu.edu.cn

**Keywords:** quantum dots, perovskite solar cells, interfacial layer, energy level match, passivate defects

## Abstract

Perovskite solar cells (PSCs) are currently attracting a great deal of attention for their excellent photovoltaic properties, with a maximum photoelectric conversion efficiency (PCE) of 25.5%, comparable to that of silicon-based solar cells. However, PSCs suffer from energy level mismatch, a large number of defects in perovskite films, and easy decomposition under ultraviolet (UV) light, which greatly limit the industrial application of PSCs. Currently, quantum dot (QD) materials are widely used in PSCs due to their properties, such as quantum size effect and multi-exciton effect. In this review, we detail the application of QDs as an interfacial layer to PSCs to optimize the energy level alignment between two adjacent layers, facilitate charge and hole transport, and also effectively assist in the crystallization of perovskite films and passivate defects on the film surface.

## 1. Introduction

In the past, the development of human society was largely based on the exploitation and use of large amounts of fossil energy, which is becoming increasingly depleted as mankind overexploits it [1]. At the same time, the environmental problems caused by this are a constant source of concern, making the use of clean and renewable energy an urgent priority [2,3]. Solar energy is an inexhaustible source of energy that has unique advantages among many clean energy sources and has therefore received a great deal of attention from researchers [4,5].

**Figure 1:**
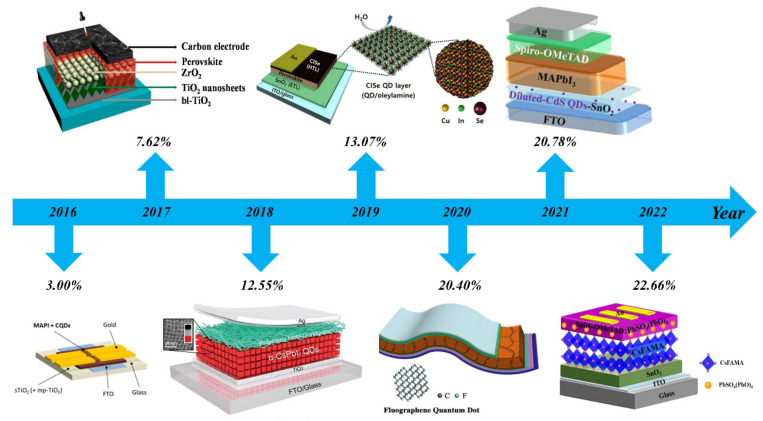
Annual progress of PSCs from 2016 to 2022. Power conversion efficiency, device structure, and major breakthroughs of different types of device are illustrated [39,40,41,42,43,44,45]. Reproduced from [39,40,41,42], with permission from Elsevier, 2016, 2017, 2018, 2019. Reproduced from [43,44,45], with permission from American Chemical Society 2020, 2021, 2022.

Photovoltaic power generation as an important use of solar energy has received continuous and steady development. Photovoltaic power generation mainly uses solar cell devices, of which PSCs are developing particularly rapidly, to convert solar energy into electricity [6,7,8]. Over the last decade, the PCE of PSCs has improved from 3.8% to 25.5%, a sixfold increase and the highest among thin-film solar cells [9], which is shown in Figure 1. Despite their many advantages, PSCs suffer from the inability to deposit perovskite films over large areas [10,11,12], poor stability [13,14,15,16,17], the tendency of perovskite to undergo phase changes [18,19,20], and severe hysteresis in formal structures [21,22,23], which are key issues that severely limit the industrial application of PSCs. As a consequence, researchers have been searching for some time for ways to optimize PSCs with strategies such as interfacial modifications [24,25,26,27], elemental doping [28,29,30,31], material substitution [32,33,34,35,36], etc. QDs are popular materials today and are favored by researchers due to their properties such as quantum size effect and surface effect [37,38]. After using QDs as an interfacial modification layer for PSCs, researchers found that the energy level arrangement was optimized between the different layers and facilitated charge transport, while QDs provided nucleation sites for perovskite, aiding its crystallization and the formation of finer and more numerous grains. In this article, we review the recent research progress on QDs as an interface modification material for PSCs and provide a detailed analysis of the role of different QDs as an interface modification layer in PSCs. Finally, we make our own suggestions for the future application of QDs as an interface modification layer in PSCs.

## 2. Structure of PSCs

**Figure 2:**
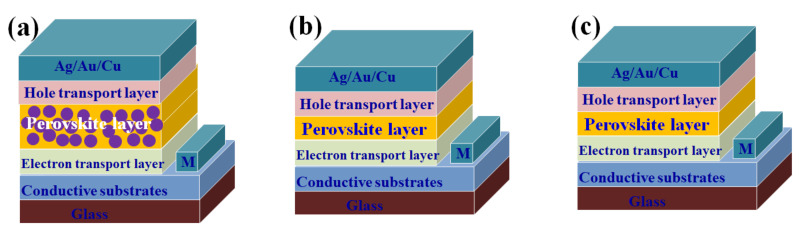
(**a**) mesoporous upright structure; (**b**) upright structure; (**c**) inverted structure.

PSCs are a new type of thin-film solar cells whose structures can be divided into formal (n-i-p) and inverted (p-i-n) structures (Figure 2) [46]. Formal structures include both formal mesoporous structures and formal planar structures. The basic structure of formal mesoporous PSCs is fluorine-doped tin oxide (FTO)/compact TiO_2_ (c-TiO_2_)/mesoporous electron transport layer (ETL)/perovskite/hole transport layer (HTL)/metal electrodes (Au or Ag). ETL is generally SnO_2_, TiO_2_, ZnO, C_60_ (buckminsterfullerene), and BCP (bathocuproine). HTL is generally CuSCN, PEDOT:PSS (poly(4-butylphenyldiphenylamine), poly(3,4-ethylenedioxythiophene) polystyrene sulfonate), PTAA (polytriarylamine), and Spiro-OMeTAD (2,2′,7,7′-tetrakis-(N,N-di-4-methoxyphenylamino)-9,9′-spirobifluorene). Formal planar structures are the simplest of the PSCs, but formal planar structures generally suffer from hysteresis effects, a drawback for which formal planar structures have been criticized [23,47].

The basic structure of the inverted planar PSCs is FTO/HTL/perovskite/ETL/metal electrodes [48,49]. Hysteresis effects have been reported to be significantly weaker for PSCs with inverted structures than for those with formal structures, and the corresponding filling factor (FF) exceeds 0.8, higher than for most cells with formal structures [50,51,52,53]. However, the PCE of inverted PSCs is still lower than that of conventional PSCs.

The operation of PSCs can be divided into three parts: generation, collection, and transport of charge and holes. In the formal structure, when the PSCs receive sunlight, the perovskite layer first absorbs the photons and produces electron–hole pairs. The uncompounded electrons and holes are then collected by the ETL and HTL, respectively, with the electrons eventually being collected by the conductive glass FTO and the holes by the metal electrode. When the FTO is connected to a metal electrode, the circuit begins to operate, producing a photocurrent. The current direction of the PSCs in the inverted structure is the opposite of the formal one; holes flow towards the pre-conducting glass and electrons flow towards the metal electrode.

## 3. Introduction of QDs

QDs are semiconductors that are nanoscale in all three dimensions of the material in three-dimensional space, mostly prepared by thermal injection and low-temperature solution methods [6,37], and have been widely used in solar cells [54,55,56,57], photocatalysis [58,59,60], displays, and light-emitting diodes [61,62,63,64,65,66]. Quantum size effect and multi-exciton effect are important properties of QDs. The band gap of QDs can be fine-tuned by nanoscale modulation, which can make optical absorption edge cover the infrared visible spectrum and improve light harvesting. In addition, typical photovoltaic materials merely generate one electron–hole pair after absorbing one photon with sufficient energy. However, due to the multi-exciton effect of QDs, more than one electron–hole pair can be activated by each photon in QDs. As a result, they are widely used in PSCs because of their tunable energy bands and ease of preparation. Examples include TiO_2_ QDs, ZnO QDs, and SnO_2_ QDs [32,35,67,68], which are non-toxic, cost-effective, and have high charge mobility. PbS QDs have a large exciton Bohr radii, and they are able to absorb near-infrared light, enhancing the PCE of PSCs. In addition, carbon quantum dots (CQDs), graphene quantum dots (GQDs), and black phosphorus quantum dots (BP QDs) are the most widely used QDs, and they have great potential for application not only as additives and interfacial layers but also as electron transport materials (ETM) and hole transport materials (HTM), hence the higher stability of the obtained PSCs.

When QDs are used as an interfacial layer, the optimization of the energy level alignment is more dramatic than that when used as an additive. Additionally, direct contact with the perovskite enables the crystallization of perovskite films to be assisted over a large area, forming a high-quality film. As a consequence, QD interface modification layers are widely used in PSCs, with great application value shown.

## 4. QD-Modified Interface Layer

In addition to the doping of ETL or HTL, interface engineering is currently an effective strategy to maximize the performance of PSCs. The ability to efficiently and rapidly transfer photogenerated carriers is crucial. During transmission, the PCE of the device is severely degraded once the carriers are trapped by defects in the perovskite, ETL, or HTL. To promote charge transfer, improve perovskite crystallization, and retard carrier complexation, researchers have introduced QDs extensively at the perovskite/ETL and perovskite/HTL interfaces. At the same time, the introduction of an interfacial layer can be used as a separating layer to improve the stability of PSCs in the environment on one hand and to reduce current hysteresis caused by charge trapping or ion migration on the other hand [69,70].

### 4.1. QD-Modified Perovskite/ETL Interface

When used as a layer to modify the perovskite/ETL interface, QDs are able to optimize the energy level alignment between the two layers and facilitate charge transport. On this basis, some QDs can also be used as nucleation centers to assist in the crystallization of perovskite.

#### 4.1.1. CQD-Modified Perovskite/ETL Interface

**Figure 3:**
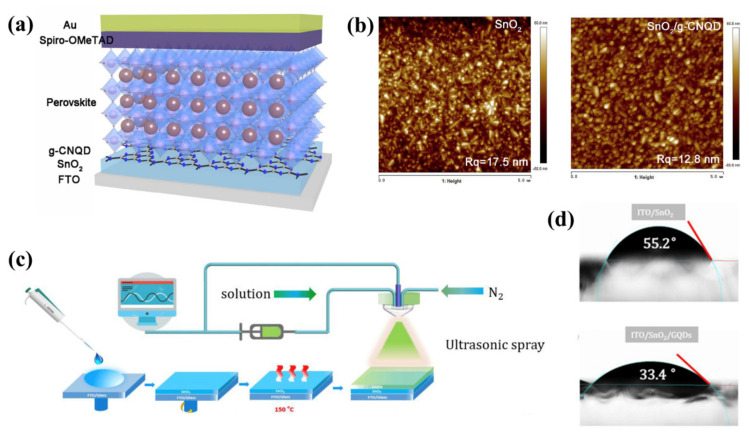
(**a**) Schematic representation of the structure of PSCs with graphdiyne carbon nitride QDs as an interfacial layer. Reproduced from [77], with permission from Elsevier, 2020. (**b**) AFM images of pristine SnO_2_ film and SnO_2_/g-CN QD film. Reproduced from [77], with permission from Elsevier, 2020. (**c**) Schematic diagram of GQDs deposited by ultrasonic atomization. Reproduced from [27], with permission from Elsevier, 2019. (**d**) Comparison of the water contact angles of SnO_2_ and SnO_2_/GQD films. Reproduced from [27], with permission from Elsevier, 2019.

CQDs, which are typically <10 nm in size, are the most widely used QD material today [71]. Their low cost and excellent optoelectronic properties have made them a popular choice for researchers [72,73,74,75]. In 2018, Ding et al. [76] inserted CQDs between TiO_2_ and CsPbBr_3_ to act as an electronic bridge and facilitate charge transport. The final all-inorganic PSCs achieved a PCE of 7.86%. Despite the low PCE compared to organic PSCs, this provided a paradigm for the realization of optimized all-inorganic PSCs. In 2020, Liu et al. [77] prepared graphitic carbon nitride quantum dots (g-CN QDs) and used them as the SnO_2_/perovskite interface through spin-coating deposition (Figure 3a). The intrinsic cross-link ability of g-CN QDs made for a low number of grain boundaries and traps, which facilitated charge transport. It also allowed the SnO_2_ film to be smoother, with a dramatic reduction in the root mean square roughness of the film from 17.5 nm to 12.8 nm (Figure 3b). Moreover, an impure PbI_2_ peak (2θ = 12.7°) can be founded in the SnO_2_/perovskite film while being undistinguished in the SnO_2_/g-CN QDs/perovskite film; this result confirmed that the uniform and smoother perovskite films with higher phase purity were achieved by g-CN QD modification. Therefore, the final PCE was as high as 21.23%, and the PCE of the g-CN-QD-modified device retained over 90% of its initial performance after 30 days.

#### 4.1.2. GQD-Modified Perovskite/ETL Interface

In 2014, Zhu et al. [24] inserted an ultra-thin graphene GQD layer between perovskite and mesoporous TiO_2_. The rate of electron extraction at the interface was accelerated, and the PCE of the PSCs increased to 10.15%, significantly higher than the efficiency of 8.81% without the introduction of GQDs. In 2017, Ryu et al. [25] prepared GQDs of 7 nm, 10 nm, and 14 nm by chemical oxidation and carbon brazing dimensional cutting. The quantum size effect was then used to successfully regulate the energy band energy of GQDs and optimized energy level arrangement in PSCs. The absorbance in the long wavelength region was enhanced with the increasing size of the GQDs. In addition, optical images of the 14 nm GQDs, 10 nm GQDs, and 7 nm GQDs under a 364 nm UV lamp revealed different colors, which were yellow, green, and blue, respectively. The lowest unoccupied molecular orbital (LUMO) of 10 nm and 14 nm GQDs lies between TiO_2_ and perovskite, so it can effectively promote electron transfer from perovskite to ETL. Additionally, 7 nm GQDs have a higher LUMO energy level, which is not conducive to charge transfer. The final perovskite solar cell quoting 14 nm GQDs showed the highest PCE, which was 19.11%. There was also a significant reduction in hysteresis. The reduction ratio of PCE at forward scan to that at reverse scan (=(PCE_rev_ − PCE_for_)/PCE_rev_) of 14 nm GQDs was 18.63%, which was a lower value compared with that of the reference cells (=27.13%).

Due to the similar solvent polarities of the low-temperature SnO_2_ and GQDs solutions, it is exceptionally difficult to deposit GQDs on a SnO_2_ crystal layer using the conventional spin coating method. Moreover, the component of GQDs in SnO_2_ is tiny; it was difficult to control the concentration of GQDs accurately, and excess GQDs would lead to dramatic drop in efficiency. Accordingly, exploring an accurate controlled method was essential. In 2019, Xia et al. [27] used ultrasonic atomization to deposit an ultra-thin layer of GQDs between the perovskite and SnO_2_ (Figure 3c), which is a solution based, scalable, open-air technology. In this process, GQDs were dissolved in solvent and ultrasonically atomized into mist droplets with a radius of several micrometers, which were carried out by inert gas flow and deposited onto substrates. As a result, the ultrathin GQDs layer effectively prevented damage to the SnO_2_ surface’s chemistry by aqueous solutions. The SnO_2_ films were more compacter with the introduction of GQDs. At the same time, the surface energy and contact angle of the SnO_2_ films were significantly reduced, facilitating the diffusion of the perovskite precursor solution on the SnO_2_ film (Figure 3d), which resulted in better contact between the SnO_2_ and the perovskite film. The work functions of SnO_2_ and SnO_2_/GQDs were calculated to be −4.35 eV and −4.23 eV, respectively. The decrease in work function of SnO_2_ film by GQDs was mostly derived from the formation of dipoles on the surface, which also led to a shift in the ohmic contact between perovskite and SnO_2_, enabling barrier-free electron extraction from perovskite to SnO_2_, with a final PCE of 16.54%.

**Figure 4:**
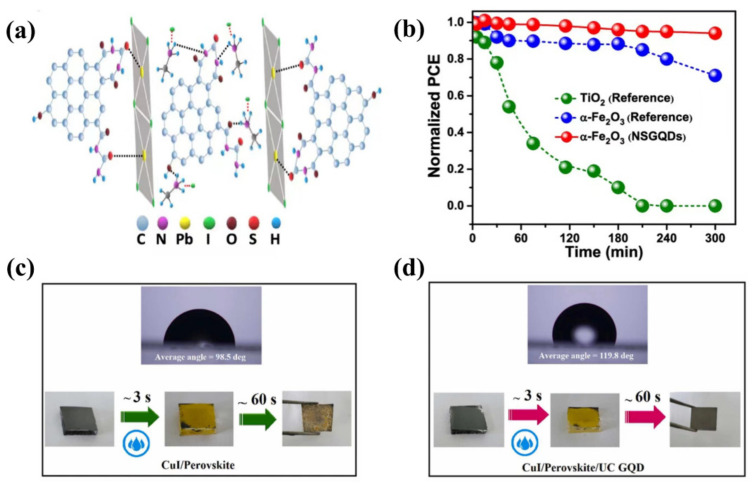
(**a**) Schematic representation of the interaction between PbI_2_-MAI and NSGQDs. Reproduced from [78], with permission from American Chemical Society, 2020. (**b**) Normalized PCE decay of perovskite devices based on different ETLs as a function of UV irradiation time at an intensity of 500 mW cm^−2^. Reproduced from [78], with permission from American Chemical Society, 2020. (**c**,**d**) CuI/Perovskite and CuI/Perovskite/UC GQD films. Optical images of the perovskite films (without encapsulation) as a function of humidity exposure time in 90 ± 5% RH. Reproduced from [80], with permission from Elsevier, 2021.

Compared to TiO_2_ as the ETL, the degradation of performance under long-term light exposure is effectively prevented when using α-Fe_2_O_3_ as the ETL. However, the PCE was still low due to poor contact between the ETL and perovskite. Chen et al. [78] found that N,S-co-doped graphene quantum dots (NSGQDs) had many functional groups (C=O,C=S,C-N,C-O) which can optimize the performance of PSCs based on α-Fe_2_O_3_ ETL. NSGQDs as an electronic interface modification layer effectively promoted charge separation and inhibited charge complexation. When NSGQDs were used as the hole transport interface modifier layer, they were also able to optimize the energy band between perovskite and HTL. In addition, NSGQDs were added to the MAPbI_3_ film to increase the grain size and passivate the surface defects of the perovskite film (Figure 4a). The calculated conductivity of 0.04 mS cm^−1^ for the perovskite modified by QDs was three times higher than the pristine perovskite, for which conductivity was 0.013 mS cm^−1^. As a result of the multi-interface engineering, the PCE had thus increased from 14% to 19.2%. In addition, the ionic diameter of iodide (0.412 nm) was larger than that of the graphene lattice (0.246 nm); the iodide ions in the perovskite could not permeate the two-dimensional crystal lattice of graphene. The GQDs layer prevented the diffusion of iodide ions to the metal electrode side. NSGQDs can effectively prevent thermal degradation, causing PSCs to preserve up to 90% of their initial PCE after being stored in darkness at 85 °C for 300 h. The pristine PSCs only retained 45% of their initial efficiently. Therefore, the heat stability of the device with NSGQDs significantly increased. (Figure 4b). In 2021, Gao et al. [79] used imidazole-bromide-functionalized GQDs (I-GQDs) as an interfacial modification layer to modulate the performance of planar PSCs. The incorporation of I-GQDs not only reduced the interface defects for achieving a better energy level alignment between ETL and perovskite but also improved the film quality of FAPbI_3_ perovskite, including enlarged grain size, lower trap density, and a longer carrier lifetime. The PCE of the planar PSC was 22.37%. It appeared that functionalized GQDs can effectively passivate interfacial defects and optimize the energy level alignment of the interface, offering additional advantages and properties compared to single GQDs. This provides an effective way to optimize QDs.

Photonic up-conversion was recognized as an effective interface modification method due to its ability to reduce spectral mismatch losses. In 2021, Irannejad et al. [80] thought in terms of the up-conversion properties of QDs, up-converting graphene quantum dots (UC GQDs) were prepared. UC GQDs have a wide optical absorption range and are well adapted to perovskite absorption spectra [81,82], UC GQDs enhanced light trapping capacity of the perovskite layer, with a PCE of 19.79%. The most striking feature is the improved hydrophobicity of the device. CuI has a contact angle of up to 119.8° with the perovskite film. In addition, when the UC GQDs layer was introduced, the structure of perovskite film was capable of self-healing (Figure 4c,d). The surface of perovskite films was prone to decomposition when water was absorbed, and the decomposed film appeared yellow. The interconnected grain boundaries of UC GQDs can effectively suppress escaping CH_3_NH_3_^+^ (MA^+^) and CH_2_(NH_2_)_2_^+^ (FA^+^) ions. After removal of the water vapor, the perovskite returned from its degraded state to its original structure again. This process was an effective way to facilitate the commercialization of PSCs. All details are displayed in Table 1 [24,25,26,27,76,77,78,79,80,83].

#### 4.1.3. PQD-Modified Perovskite/ETL Interface

Of all the QDs that can optimize the ETL/perovskite interface, perovskite quantum dots (PQDs) the most pronounced specificity, as they inherently have a similar structure to perovskite materials. Because of the limitations of the PQD preparation process, most PQDs are currently used in inverted PSCs, where they can improve crystallization of perovskite films and passivate defects on perovskite surfaces.

**Figure 5:**
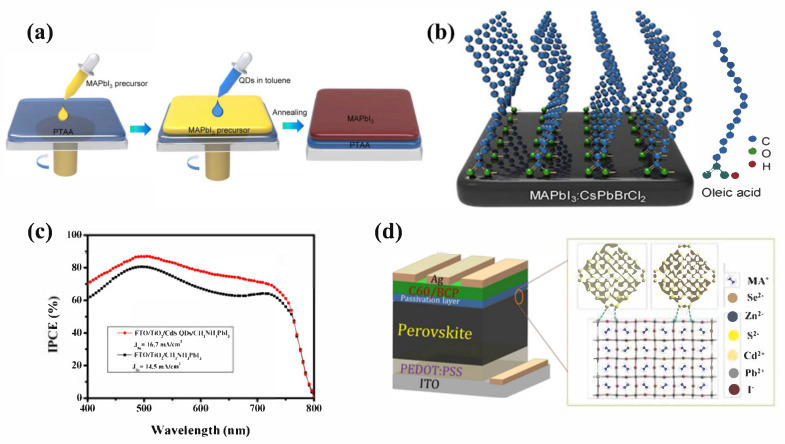
(**a**) Schematic illustration of the procedure for preparing MAPbI_3_ film by using CsPbBrCl_2_ QDs as an anti-solvent suspension. Reproduced from [84], with permission from Elsevier, 2019. (**b**) Schematic representation of the uniform distribution of elements in MAPbI_3_ film and the self-assembly of OA molecules on the surface of MAPbI_3_ film. Reproduced from [84], with permission from Elsevier, 2019. (**c**) IPCE spectra of TiO_2_/MAPbI_3_ (black line) and TiO_2_/CdS/MAPbI_3_ (red line) solar cells. Reproduced from [93], with permission from Elsevier, 2018. (**d**) Schematic of the structure of CdSe/ZnS QDS-based device. Reproduced from [95], with permission from Royal Society of Chemistry, 2020.

In 2019, Zheng et al. [84] dispersed CsPbBr_2_Cl QDs in toluene as an anti-solvent and prepared an active layer of MAPbI_3_ by low-temperature one-step inverse solvent extraction (Figure 5a). The QDs partly act as nucleation agents, thus promoting the growth of a more uniform perovskite film. Self-assembly of oleic acid (OA) ligands introduced by QDs in a toluene solvent exposes the hydrophobic chains at the interface and thus enhanced the hydrophobicity of the perovskite film surface; contact angle increased from 64.5° to 88.8°, and the stability of the device in humid environments improved. When QDs met a wet MAPbI_3_ precursor solution, the PQDs decomposed, doping the elements within the MAPbI_3_ membrane and leaving the ligands on the surface of the MAPbI_3_ membrane (Figure 5b). Cs, Br, and Cl elements were uniformly distributed throughout the film. The adhesions on the surface of the film effectively passivated the defects at the interface. They also compared the Fourier transform photocurrent spectroscopy (FTPS) signals below 1.5 eV for the PSCs with and without QDs. The FTPS signal from the device with QDs was observed to be lower at the energies from 1.2 to 1.5 eV than those of the pristine device, which showed that QDs mitigated the energy disorder of MAPbI_3_, narrowed the band tail electronic states, and reduced the mid-gap states of MAPbI_3_. Finally, the device-based MAPbI_3_ treated with CsPbBrCl_2_ QDs achieved a PCE of 21.5% and maintained 80% of initial performance. In contrast, the pristine device without CsPbBrCl_2_ QDs dramatically degraded to 27% of its initial efficiency. PQDs are well-optimized for inverted PSCs.

In the same year, Yang et al. [85] prepared CsPbBr_3_ QDs through a modified emulsion; they tested the response of the device at low magnetic excitation strengths. Hence, the addition of perovskite QDs could reduce the surface potential of perovskite films and mitigate the non-uniformities in their surface potentials. Such a change in the morphology and surface laterally may contribute to the suppression of non-radiative recombination on surface regions of perovskite films. In addition, they calculated that the coherence lengths of the crystalline lattice were ~62 nm for both the control and CsPbBr_3_ QDs films with the Debye-Scherrer equation, which meant that the introduction of CsPbBr_3_ QDs did not distort the crystal structure of the three-dimensional perovskite films and they matched well. Consequently, CsPbBr_3_ QDs make the device highly efficient; the final PCE was obtained as 21.03%, and the PCE of the device without CsPbBr_3_ QDs was 19.08%. All details are displayed in Table 2 [84,85,86].

#### 4.1.4. Other-QD-Modified Perovskite/ETL Interface

PbS QDs have been used for interfacial modifications due to their large exciton Bohr radius and near-infrared absorption capabilities. In 2015, Yang et al. [87] prepared PbS QDs using the successive ionic layer absorption and reaction (SILAR) technique, and the device containing the interfacial layer of PbS QDs showed remarkably enhanced light absorption in the 330 to 1400 nm range. The catalytic decomposition of TiO_2_ under UV light was also reduced, resulting in a final PCE of 4.92%.

Cd-based QDs were used in PSCs due to their specific properties as a special quantum confinement effect, polaronic effects, and interaction with the local environment [88,89,90,91]. Compared to other QDs, CdS QDs have good stability and special quantum confinement effects [92]. Ali et al. [93] used the SILAR technique to prepare CdS QDs and used them as an interfacial layer; PCE increased from 8.93% to 10.52%. As shown in Figure 5c, the induced-photon-to-current efficiency (IPCE) spectra showed an improvement of induced photons to current conversion by doping with CdS QDs. On this basis, Yenel et al. [94] used the two-phase method to synthesize colloidal CdS QDs, which were used as a modifier between ETL and perovskite. A comparison was made between oleic-acid-covered and pyridine-covered CdS QDs. Due to the long hydrocarbon chain of oleic acid, which prevented the injection of electrons, a decrease in PCE was observed. On one hand, pyridine passivated the surface defects of CdS QDs because of its molecular structure; on the other hand, because of the coordination of pyridine with uncoordinated Pb^2+^ ions, the crystalline quality of the perovskite films was improved, increasing PCE from 11.7% to 13.2%.

Additionally, CdSe/Zns hybrid QDs have been found to improve the performance of PSCs. In 2019, Hanmandlu et al. [95] deposited CdS/ZnS QDs and CdSe/ZnS QDs on MAPbI_3_ films as a passivation layer to modify the perovskite/C_60_ interface (Figure 5d). Due to the strong binding between S^2−^, Se^2−^, and Pb^2+^ ions in the perovskite, QDs were uniformly distributed on the surface of the perovskite. His team, through density functional theory (DFT), found stronger interactions between perovskite and CdSe/ZnS QDs because Se^2+^ ions have lower electronegativity than S^2−^ ions. Thus, CdSe/ZnS QDs can passivate surface defects and grain boundary traps in perovskite films more effectively, resulting in a final max PCE of 19.89%, which also reduced hysteresis and improved stability of the device to some extent.

The use of QDs as an interfacial layer can effectively facilitate electron transport, but still presents a remarkable challenge in terms of enhancing light-harvesting capabilities. For this reason, in 2017, Gao et al. [96] used CuInS_2_ QDs as an interface between TiO_2_ nanorods and perovskite. CuInS_2_ QDs have a high absorption coefficient (c.a. 10^5^ cm^−1^), and therefore, the devices had a significantly extended absorption range of 300 to 800 nm. The band gap of CuInS_2_ QDs is approximately 1.6 eV, which effectively optimized the energy level alignment between the two layers and facilitated charge transport, resulting in a PCE of 11.70% for the final device based on CuInS_2_ QDs as the interface. In the following time, they prepared CuInS_2_ QDs in the size range of 1 nm to 3 nm in 2019 [97], which were again used as an interface between TiO_2_ nanorods and perovskite. XRD showed that CuInS_2_ QDs strongly influence the crystallization of perovskite, promoting the growth of CH_3_NH_3_PbI_3_ crystals along the (312) crystal plane. The CuInS_2_-QD-based device showed good performance and retained 41% of its initial PCE after 30 days of standing in the environment; the device without CuInS_2_ QDs retained only 12% of its original efficiency. This was a major breakthrough for CuInS_2_ QDs as an interfacial layer.

Researchers have also attempted to use rare earth elements in PSCs, but little has been reported so far. In 2016 He et al. [98] produced homogeneous NaYF_4_:Yb/Er QDs for the first time and used them as an interfacial layer. NaYF_4_:Yb/Er QDs enabled PSCs to absorb low-energy photons near infrared solar photons more efficiently and converted them into high-energy photons, thereby generating additional photocurrents. The PCE of the device was increased significantly and reached 18.1%. This experiment provided an effective way for PSCs to minimize the loss of non-absorbed energy and facilitated PSCs breaking the Shockley-Queisser limit. All details are displayed in Table 3 [87,93,94,95,96,97,98,99].

### 4.2. QD-Modified Perovskite/HTL Interface

QDs are currently used as an interface layer between perovskite/HTL to passivate film defects, optimize energy level alignment, and assist in the crystallization of the perovskite layer, thus improving the stability of the equipment.

#### 4.2.1. CQD- and GQD-Modified Perovskite/HTL Interface

Currently CQDs and GQDs are less reported as the perovskite/HTL interface layer.

However, studies have been carried out by researchers. In 2017 Zhou et al. [100] prepared the first thin films of CQD-sensitized CsPbBr_3_ perovskite inverse opal (IO) with improved light utilization, culminating in a PCE of 8.29% for the all-inorganic PSCs they fabricated. In 2019, Wei et al. [101], using H_2_SO_4_ intercalation and NH_3_ stripping, synthesized ultra-fine flake graphitic carbon nitride QDs (E-g-C_3_N_4_ QDs) and used them as an interface layer between perovskite and HTL. E-g-C_3_N_4_ QDs can form hydrogen bonds on the grain boundaries of the film. These bonds are easily identified and located on the grain boundaries for the purpose of passivating defects on the film surface. The PCE obtained was 15.8%, which was a 35% improvement over the reference cell. In 2022, Khorshidi et al. [102] applied hydrophobic GQDs (HGQDs) to PSCs. The amide groups contained within the HGQDs uncoordinated Pb^2+^ ions to passivate the film surface and reduce the defect density, with a PCE of 18.30%. At the same time, the hydrophobic alkyl group made the perovskite film impermeable to water, significantly improving stability. All details are displayed in Table 4 [100,101,102].

#### 4.2.2. PQD-Modified Perovskite/HTL Interface

In 2016, Cha et al. [103] synthesized of CH_3_NH_3_PbBr_3−x_I_x_ QDs and used them as a modification interface layer. There was little difference in the stability of PSCs with and without the addition of CH_3_NH_3_PbBr_3−x_I_x_ QDs. However, the valence band edge of CH_3_NH_3_PbBr_0.9_I_2.1_ QDs (−5.40 eV) lies between the valence band of perovskite (−5.43 eV) and the highest molecular orbital of HTM (−5.22 eV), with a remarkable increase in the interfacial hole transfer rate. The PCE was increased from 10.34% to 13.32%. This work provided an example and mechanism for subsequent interface commissioning.

**Figure 6:**
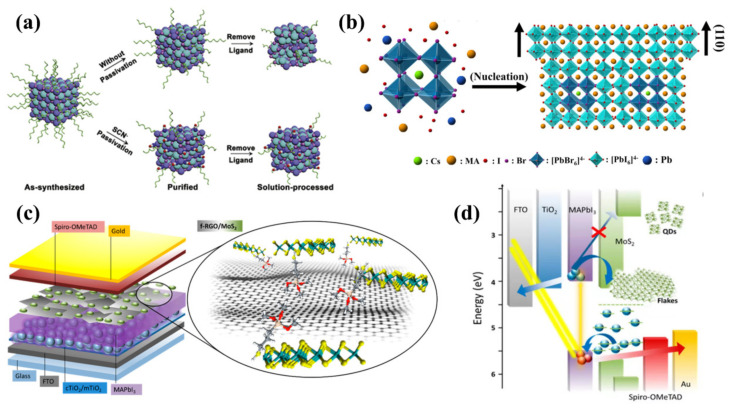
(**a**) Schematic comparing the CsPbI_3_ QDs with and without short-chain thiocyanate anion (SCN^−^) capping after removing the ligand chain on the QD surface using saturated Pb(OAc)_2_ ethyl acetate (EA) solution. Reproduced from [104], with permission from Elsevier, 2018. (**b**) Schematic diagram of the nucleation mechanism of MAPbI_3_ induced with CsPbBr_3_ QDs. Reproduced from [111], with permission from IOP Science, 2020. (**c**) Schematic of the structure of a mesoporous PSC based on MoS_2_ QDs. Reproduced from [115], with permission from American Chemical Society, 2018. (**d**) Schematic diagram of the energy band structure of a PSC with MoS_2_ QDs as an interfacial layer. Reproduced from [115], with permission from American Chemical Society, 2018.

CsPbI_3_ is used as one of the suitable interfacial materials in all-inorganic perovskite materials with high valence band position (VBP) and high stability. In 2018, Bian et al. [104] prepared CsPbI_3_ QDs; they replaced Pb^2+^ ions with Mn^2+^ ions, then capped with long-chain oleic acid and short-chain thiocyanate anion (SCN^−^). Strong chemical interactions occurred between the SCN^−^ and QD surfaces, which formed Pb-S and N-H bonds, effectively preventing the clustering of QDs (Figure 6a). They then modified the surface of CsPbI_3_ QDs using the formamidinium iodide (FAI) solution to improve the charge transport capacity. The band gap of CsPbI_3_ is 1.77 eV and that of CsPbBrI_2_ is 1.91 eV; both are low and close to each other, resulting in an increase in the light absorption capacity of the film. In addition, the CsPbBrI_2_/CsPbI_3_ QDs interface forms a strong component-graded heterojunction due to Br^−^ and I^−^ ion exchange, facilitating charge extraction from the device. The final PCE was 14.45% for the latter CsPbBrI_2_- and CsPbI_3_-QD-based device. Liu et al. [105] used Cs_2_Ac and PbI_2_; cubic CsPbI_3_ QDs in the size range of 9–13 nm were synthesized by shape modulation with ethyl acetate. Then, they deposited them onto FAMAPbI_3_, significantly improving the long-term stability of the device. The initial PCE of the device without the CsPbI_3_ QDs was almost entirely lost after 35 days, corresponding to an attenuation of 100%. In contrast, the device with the CsPbI_3_ QDs experienced a PCE decrease from 100% to 80% after 35 days. Finally, the PCE of the champion device reached 18.56%.

Inorganic CsPbI_2_Br QDs are of increasing interest due to their excellent phase stability and optoelectronic properties [106,107]. Zhang et al. [108] prepared size-graded heterojunction PSCs by mixing CsPbI_2_Br and CsPbI_2_Br QDs whose surfaces were covered with organ iodized salts for the first time. The graded combination not only optimized the surface of the QDs, but also the energy level alignment between CsPbBrI_2_ and HTL, facilitating the extraction and transport of holes between the two layers. The profile structure enabled a continuous upward shift in energy levels, which resulted in a short-circuit current (Jsc) of 12.93 mA cm^−2^ and a significant increase in PCE to 12.39%, the highest record for CsPbBrI_2_-based PSCs at that time.

In 2019, Akin et al. [109] used ultra-thin CsPbBr_1.85_I_1.15_ QDs as an interface layer between perovskite and Spiro-OMeTAD. The inorganic PQDs passivated the defects at the interface and significantly inhibited carrier compounding at the interface. As a result, cells with this interfacial layer were free of hysteresis, with a PCE of 21.14%. In addition, the presence of a FRET mechanism at the interface due to the addition of PQDs reduced the decay time from 5.77 ns to 4.66 ns. CsPbBr_1.85_I_1.15_ QDs demonstrated excellent humidity resistance; the device with QDs can retain more than 90% of its initial efficiency after 30 days in a high humidity environment, significantly better than other devices without CsPbBr_1.85_I_1.15_ QDs.

In 2019, Que et al. [110] prepared Cs_1−x_FA_x_PbI_3_ QDs in n-hexane using a cation exchange method. Among the various quantum dots that were used, the Cs_0.57_FA_0.43_PbI_3_ QDs exhibited the best performance. The introduction of QDs enriched the surface of FAPbI_3_ films with Cs^+^ ions, which remarkably inhibited the decomposition of perovskite. This enhanced phase stability, ultimately achieving a PCE of 20.82%. In 2020, Yao et al. [111] introduced 0.001 mg/mL CsPbBr_3_ QDs into PSCs, and the QDs provided non-uniform phase-shaped nucleation centers for MAPbI_3_ films, allowing the (110) crystal plane to be denser (Figure 6b). Pinholes on the film surface were effectively eliminated, reducing surface roughness and avoiding carrier capture by defects on the film. The PCE of the final champion device was 20.17%. All details are displayed in Table 5 [103,104,105,108,109,110,111,112].

#### 4.2.3. Other-QD-Modified Perovskite/HTL Interface

PbS QDs had been mentioned previously as a perovskite/ETL interface for PSCs and reported as a perovskite/HTL interface modifier. In 2019, Zhu et al. [113] used narrow-bandgap PbS QDs as an interface modification material. The cubic structure of the PbS QDs can interconvert with the tetragonal MAPbI_3_ and passivate the defects on the MAPbI_3_ surface. However, when the concentration of PbS QDs is too high, they will accumulate on the film and moisture corrosion will occur, causing the roughness of the film to rise, which was not conducive to the extraction of cavities. In particular, PbS QDs can absorb near-infrared light, contributing to an increase in PCE from 17.46% to 19.24%. However, this result was still low compared to the results of another study. When 4-hydroxybenzaldehyde (HBA) was used as the interface modification layer, the champion device achieved an efficiency of 20.89% [114]. There was still a gap between them.

Najafi et al. [115] obtained MoS_2_ QDs by liquid phase exfoliation and used them as interfacial modification materials (Figure 6c). Due to the quantum size effect, the optical band gap of MoS_2_ was increased from 1.4 eV for the flakes to >3.2 eV for QDs, raising the minimum energy of its conduction band (from −4.3 eV for the flakes to −2.2 eV for QDs) above one of the conduction bands of MAPbI_3_, which was between −3.7 and −4 eV. It has been shown that zero-dimensional MoS_2_ QDs enabled better energy band alignment (Figure 6d), which enhanced hole extraction but limited electron transport to a certain extent [116,117,118]. Additionally, after doping with reduced graphene oxide (RGO), the pinholes on the MoS_2_ QDs were blocked by RGO because of its two-dimensional properties. The final PCE of the PSCs with MAPbI_3_ as the substrate reached 20.12%. In another study, Mateus Torres-Herrera et al. [119] used MoO_x_:Au composite coatings with thicknesses of 10–20 nm. However, the PCE of the champion device was 12.87%, significantly lower than MoS_2_ QDs as an interfacial layer modified device. The reason for this may be a mismatch in the energy level alignment between the MoO_x_:Au composite coatings and the perovskite layer, resulting in slow hole transport. Thus, QDs seem to be superior among many interfacial modification materials.

**Figure 7:**
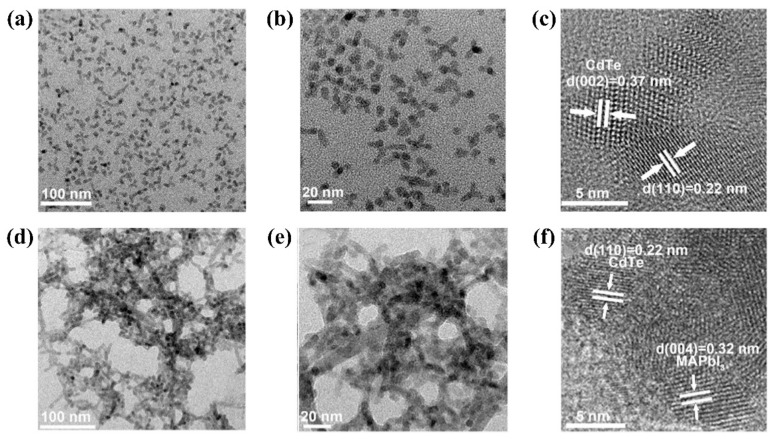
(**a**) The TEM, HRTEM images of the CdTe QDs before (**a**–**c**) and after (**d**–**f**) ligand exchange. Reproduced from [120], with permission from Elsevier, 2018.

**Figure 8:**
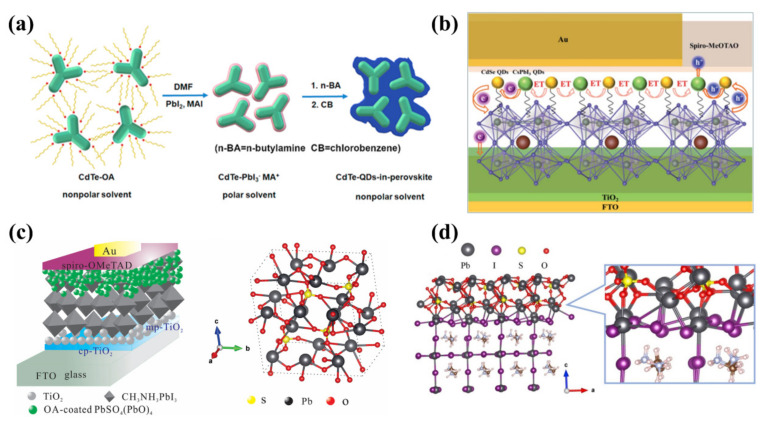
(**a**) Schematic illustration of the ligand exchange process of CdTe QDs. Reproduced from [120], with permission from Elsevier, 2018. (**b**) Schematic diagram of the energy transfer (ET) and charge transfer in the device. Reproduced from [121], with permission from Royal Society of Chemistry, 2020. (**c**) Schematic and energy diagrams of PbSO_4_(PbO)_4_-QD-based devices. Reproduced from [123], with permission from Elsevier, 2019. (**d**) Schematic of CH_3_NH_3_PbI_3_/PbSO_4_(PbO)_4_ QDs’ interfacial passivation. Reproduced from [123], with permission from Elsevier, 2019.

In a previous article, we already mentioned Cadmium compound QDs. Similarly, Cadmium compound QDs are now being explored as an interface between HTL and perovskite. First, in 2018, Xiao et al. [120] synthesized CdTe QDs by classical thermal injection. Transmission electron microscopy (TEM) characterization (Figure 7a,b) showed that the as-synthesized CdTe QDs displayed an irregular multi-pod morphology along with some special dots. As shown in Figure 7c, the high-resolution TEM (HRTEM) showed the lattice fringe of CdTe QDs, and the lattice fringe spacings of 0.22 nm and 0.37 nm correspond to the (110) and (002) planes of the wurtzite CdTe QDs, respectively. Then, they removed the long-chain oleic acid groups by ligand exchange (Figure 8a). Figure 7d,e showed that after ligand exchange, CdTe QDs formed a network-like structure of CdTe QDs in perovskite solids. The lattice spacing of CdTe QDs after ligand exchange was calculated to be about 0.32 nm from the HRTEM image presented in the Figure 7f, which corresponded to the (004) lattice plane of MAPbI_3_. This did not cause structural instability of the perovskite. The authors analyzed four typical capping ligands for comparison. Only the CdTe QDs with MAPbI_3_-capped top ligands showed excellent PCE, while the CdTe-OA^−^-modified device showed a PCE of only 2%. This illustrated the need to remove long-chain alkyl groups from CdTe QDs. Most importantly, the modification of CdTe QDs significantly reduced the hysteresis of the device, with an absolute PCE difference of only 0.53% in the forward and reverse directions. In addition, Ge et al. [121] innovatively hybridized CdSe QDs with CsPbI_3_ QDs, which were used as an HTL/perovskite interfacial layer. CdSe QDs and CsPbI_3_ QDs displayed overlapping excitation spectra, and there was a Förster resonance energy transfer (FRET) effect between CdSe QDs and CsPbI_3_ QDs, enabling energy transfer and enhanced light absorption, a property not present in the individual QDs. The introduction of hybrid QDs optimized the energy level arrangement and facilitates the transfer of electron and hole. The mechanism is shown in the Figure 8b. A final PCE of 17.1% was obtained, and this work demonstrates the excellent performance of the hybrid QDs, adding to the options for future interfacial modifications.

Black phosphorus QDs (BP QDs) have a high hole transport rate (300–1000 cm^2^/V/S) and are widely used QDs. In 2017, Chen et al. [122] prepared stable BP QDs by ultrasonication and centrifugation. The band gap is −5.2 eV for BP QDs and −5.1 eV for PEDOT:PSS. The energy levels were well-matched, enabling particularly significant improvements in cavity transmission rates. The best performance of the hybrid PSCs, which was 16.69%, was eventually achieved when the deposited BP QDs reached three layers. This work set the stage for more two-dimensional materials with high electronic properties and high hole transport rates to be applied in PSCs.

In 2019, Chen et al. [123] reported a bifunctional spherical OA-clad PbSO_4_(PbO)_4_ QD, which had the function of blocking moisture/oxygen and passivating the surface, for the first time (Figure 8c). Compared to the single CH_3_NH_3_PBI_3_ film, the diffraction peak’s position remained the same with the addition of QDs, while the peak intensity was enhanced; this showed that the presence of PbSO_4_(PbO)_4_ QDs did not change the crystal structure of the perovskite films but enhanced their crystallization. This was attributed to the fact that the H^+^ ions of oleic acid interacted with the I^−^ ions in CH_3_NH_3_PbI_3_ and Pb^2+^ ions with the SO4^2−^ ions in PbSO_4_(PbO)_4_ QDs to form hydrogen bonds, increasing its crystallinity. The size of the perovskite crystals in the CH_3_NH_3_PbI_3_/PbSO_4_(PbO)_4_ film became larger, varying from 200 nm to about 700 nm. (Figure 8d). The PbSO_4_(PbO)_4_ QDs created an electric field at the interface between the perovskite and Spiro-OMeTAD, facilitating the transfer of holes to Spiro-OMeTAD. PSCs based on the dual-functional PbSO_4_(PbO)_4_ QDs obtained a PCE of 20.02%, which provided a novel approach for future studies. All details are displayed in Table 6 [113,115,120,121,122,123].

**Table 6:** Details of other QDs as the perovskite/HTL interface layer.

QDs	Size	Device Structure	Voc	Jsc	FF %	PCE %	Ref.
CdTe	10~20 nm	ITO/SnO_2_/FA_0.57_MA_0.43_PbI_x_Br_y_Cl_3__−x−y_/ CdTe QDs/Spiro-OMeTAD/Au	1.05	23.46	72	17.87	[120]
CdSe/CsPbI_3_	~3 nm	FTO/TiO_2_/MAPbI_3_/ CdSe/CsPbI_3_ QDs/ Spiro-OMeTAD/Au	0.976	24.60	71	17.10	[121]
MoS_2_	~2.6 nm	FTO/c-TiO_2_/m-TiO_2_/MAPbI_3_/MoS_2_ QDs:Reduced graphene oxide/ Spiro-OMeTAD/Au	1.11	22.81	80	20.12	[115]
PbS	~3.4 nm	FTO/c-TiO_2_/MAPbI_3_/PbS QDs/ Spiro-OMeTAD/Au	1.14	23.17	72.83	19.24	[113]
PbSO_4_(PbO)_4_	2~5 nm	FTO/c-TiO_2_/m-TiO_2_/CH_3_NH_3_PbI_3_/ PbSO_4_(PbO)_4_ QDs/ Spiro-OMeTAD/Au	1.10	24.27	75	20.02	[123]
Black Phosphorus	~5.2 nm	ITO/PEDOT:PSS/BP QDs/MAPbI_3_/PCBM/Ag	1.01	20.13	80	16.69	[122]

## 5. Summary

In this review, we elaborate on the interfaces of different QDs applied to PSCs. These contain CQDs, GQDs, PbS QDs, CdS QDs, CdSe QDs, PbSO_4_(PbO)_4_ QDs, etc. When acting as an interface layer, QDs optimized the arrangement of energy levels between two adjacent layers, resulting in higher charge and hole transport rates. Some of these QDs can also assist in the crystallization of perovskite films and reduce the photocatalytic decomposition of TiO_2_. These remarkably improve the PCE and stability of the PSCs.

A few of these QDs are more advantageous than the others, with a highly matched internal structure to the perovskite structure due to the fact that they are stabilized by long-chain ligands, such as PQDs. Although clustering of QDs can be avoided compared to QDs with short-chain ligands, QDs with long-chain ligands have an inhibitory effect on charge transport. Therefore, we must optimize QDs through rational ligand exchange both to facilitate charge transport and to avoid agglomeration phenomena.

Additionally, there are many types of interface-modified QDs available, and researchers have focused their attention on commonly used QDs, with less research on some rare elements. There are almost no reports of rare element QDs as an interface layer after NaYF_4_:Yb/Er QDs were reported. Therefore, in the future we, should look to rare elements, which are an unexplored area.

Today, hot injection is the most popular method of synthesizing QDs. However, hot injection is usually complex and toxic. Additionally, it is difficult to obtain uniform QDs with high quality and good dispersity using this method, which can result in a significant decrease in the PCE of PSCs. Therefore, there is an urgent need for simple and high-yield strategies for the synthesis of stable QDs. In this article, we mentioned many methods of manufacturing QDs, such as cation exchange, liquid phase stripping, etc. All of these methods can be used to optimize QDs. In addition, microwave synthesis allows for the uniform heating of QDs, thereby controlling the crystallization rate. QDs are prepared rapidly by pulsed laser radiation, and no ligands are introduced during the preparation process to affect the crystallization of the film. Therefore, our search for QDs to optimize PSCs should be accompanied by a search for new methods of QD preparation.

We are convinced that in the future, QDs will fully exploit their enormous potential in PSCs, making it possible to achieve efficient and stable PSCs.

## Figures and Tables

**Table 1:** Details of CQDs and GQDs as the perovskite/ETL interface layer.

QDs	Size	Device Structure	Voc	Jsc	FF %	PCE %	Ref.
CQDs	~	FTO/c-TiO_2_/m-TiO_2_/CQDs/ CsPbBr_3_/carbon:PtNi NWS	1.43	6.78	80.9	7.86	[76]
GQDs	~5 nm	FTO/c-TiO_2_/m-TiO_2_/ GQDs/CsPbBr_3_/Carbon	1.46	8.12	82	9.72	[26]
GQDs	5~10 nm	FTO/c-TiO_2_/m-TiO_2_/GQDs/ MAPbI_3_/Spiro-OMeTAD/Au	0.94	17.06	64	10.15	[24]
GQDs	7~14 nm	FTO/c-TiO_2_/GQDs/MAPbI_3_/ Spiro-OMeTAD/Au	1.12	22.47	76	19.11	[25]
GQDs	~	FTO/SnO_2_/GQDs/MAPbI_3_/ Spiro-OMeTAD/Au	1.03	23.36	68	16.54	[27]
NSGQDS	~3 nm	FTO/α-Fe_2_O_3_/NSGQDs/ MAPbI_3_:NSGQDs/ NSGQDs/Spiro-OMeTAD/Au	1.03	23.50	79	19.20	[78]
I-GQDs	10 nm	ITO/SnO_2_/I-GQDs/FAPbI_3_/ PCBM:C_60_/Ag	1.073	25.42	82	22.37	[79]
UC GQDs	4~4.5 nm	FTO/CuI/ Cs_0.05_(MA_0.17_FA_0.83_)_0.95_Pb(I_0.83_Br_0.17_)_3_/ UC GQDs/PCBM/Au	1.15	20.99	82	19.79	[80]
Graphdiyne	3~5 nm	FTO/TiO_2_/GD QDs/CH_3_NH_3_PbI_3_:GD QDS/Spiro-OMeTAD:GD QDs/Au	1.12	22.48	79	19.89	[83]
Graphite carbon nitride	10~30 nm	FTO/SnO_2_/Graphite carbon nitride QDs/Cs_x_MA_y_FA_1−x−y_PbI_z_Br_3−z_/ Spiro-OMeTAD/Au	1.14	23.39	80	21.23	[77]

**Table 2:** Details of PQDs as the perovskite/ETL interface layer.

QDs	Size	Device Structure	Voc	Jsc	FF %	PCE %	Ref.
CsPbI_3_	~10 nm	ITO/PTAA/ Cs_0.05_(FA_0.83_MA_0.17_)_0.95_Pb(I_0.83_Br_0.17_)_3_ /CsPbI_3_ QDs/C_60_/BCP/Cu	1.15	22.90	78	20.60	[86]
CsPbBr_3_	~18 nm	ITO/PTAA/ Cs_0.05_(FA_0.83_MA_0.17_)_0.95_Pb(I_0.83_Br_0.17_)_3_ /CsPbBr_3_ QDs/C_60_/BCP/Cu	1.19	22.95	77	21.03	[85]
CsPbBrCl_2_	~7 nm	ITO/PTAA/MAPbI_3_/CsPbBrCl_2_ QDs/ C_60_/BCP/Copper	1.15	23.40	80	21.5	[84]

**Table 3:** Details of other QDs as the perovskite/ETL interface layer.

QDs	Size	Device Structure	Voc	Jsc	FF %	PCE %	Ref.
PbS	5 nm	FTO/TiO_2_/PbS QDs/CH_3_NH_3_PbI_3_/P3HT/Pt	0.88	6.30	49.3	4.92	[87]
CdS	4~5 nm	ITO/m-TiO_2_/CdS QDs/MAPbI_3_/ Spiro-OMeTAD/Au	0.94	16.86	64	10.52	[93]
CdS	5 nm	FTO/c-TiO_2_/CdS QDs/MAPbI_3_/ Spiro-OMeTAD/Au	0.95	20.6	54	13.2	[94]
CdSe	2.4~3.5 nm	ITO/c-TiO_2_/m-TiO_2_/CdSe QDs/MAPbI_3_/ Spiro-OMeTAD/Au	1.08	20.57	70	15.68	[99]
CdSe@ZnS	6~7 nm	ITO/PEDOT:PSS/MAPbI_3_/CdSe@ZnS QDs/C_60_/BCP/Ag	1.08	23.5	77	19.6	[95]
CuInS_2_	3~5 nm	ITO/TiO_2_ nanorod arrays/CuInS_2_ QDs/ MAPbI_3_/Spiro-OMeTAD/Au	0.98	17.60	69	11.70	[96]
CuInS_2_	1~3 nm	FTO/TiO_2_/CuInS_2_ QDs/MAPbI_3_/ Spiro-OMeTAD/Au	0.98	19.2	71	13.3	[97]
NaYF_4_:Yb/Er	16.3 nm	FTO/c-TiO_2_/NaYF_4_:Yb/Er QDs/CH_3_NH_3_PbI_3_/ Spiro-OMeTAD/Ag	1.06	23.1	73.8	18.1	[98]

**Table 4:** Details of CQDs and GQDs as the perovskite/HTL interface layer.

QDs	Size	Device Structure	Voc	Jsc	FF %	PCE %	Ref.
CQDs	~20 nm	FTO/c-TiO_2_/m-TiO_2_/ CsPbBr_3_/CQDs/ Spiro-OMeTAD/Ag	1.06	11.34	69	8.29	[100]
E-g-C_3_N_4_	20~50 nm	FTO/c-TiO_2_/CH_3_NH_3_PbI_3_/E-g-C_3_N_4_ QDs/Spiro-OMeTAD/Au	1.10	23.2	62	15.8	[101]
Hydrophobic GQDs	7 nm	ITO/SnO_2_/CH_3_NH_3_PbI_3_/HGQDs/ Spiro-OMeTAD/Au	1.10	22.27	75	18.30	[102]

**Table 5:** Details of PQDs as the perovskite/HTL interface layer.

QDs	Size	Device Structure	Voc	Jsc	FF %	PCE %	Ref.
CsPbBrI_2_	15~20 nm	FTO/TiO_2_/CsPbBrI_2_/CsPbBrI_2_ NSs/CsPbBrI_2_ QDs/PTAA/Au	1.19	12.93	80.5	12.39	[108]
CsPbBrI_2_	10~15 nm	FTO/c-TiO_2_/CsPbBrI_2_/ CsPbBrI_2_ QDs/PTAA/Au	1.22	14.51	79.6	14.12	[112]
CsPbBr_3_	~7.5 nm	ITO/SnO_2_/MAPbI_3_/CsPbBr_3_ QDs/Spiro-OMeTAD/Au	1.11	23.57	76.88	20.17	[111]
CsPbI_3_	9~13 nm	FTO/c-TiO_2_/m-TiO_2_/ MA_0.17_FA_0.83_Pb(I_0.83_Br_0.17_)_3_/ CsPbI_3_ QDs/Spiro-OMeTAD/Au	1.09	24.42	69.72	18.56	[105]
Mn-CsPbI_3_	15~20 nm	FTO/c-TiO_2_/CsPbI_2_Br/Mn-CsPbI_3_ QDs/PTAA/Au	1.20	14.45	78.7	14.45	[104]
Cs_0.57_FA_0.43_PbI_3_	10.7 nm	FTO/SnO_2_/FAPbI_3_/Cs_0.57_FA_0.43_PbI_3_ QDs/Spiro-OMeTAD/Au	1.12	24.44	76	20.82	[110]
CsPbBr_1.85_I_1.15_	~10 nm	FTO/c-TiO_2_/m-TiO_2_/ Cs_0.05_(FA_0.85_MA_0.15_)_0.95_Pb(I_0.85_Br_0.15_)_3_/CsPbBr_1.85_I_1.15_ QDs/ Spiro-OMeTAD/Au	1.14	23.42	79	21.14	[109]
MAPbBr_0.9_I_2.1_	~5 nm	FTO/c-TiO_2_/MAPbI_3_/ MAPbBr_0.9_I_2.1_ QDs/ Spiro-OMeTAD/Cr/Au	0.95	19.51	72	13.32	[103]

## Data Availability

Not applicable.
